# Sex Difference in the Case Fatality of Older Myocardial Infarction Patients

**DOI:** 10.1093/gerona/glab152

**Published:** 2021-05-28

**Authors:** Ville Kytö, Maria Nuotio, Päivi Rautava

**Affiliations:** 1 Heart Center, Turku University Hospital and University of Turku, Finland; 2 Research Center of Applied and Preventive Cardiovascular Medicine, University of Turku, Finland; 3 Center for Population Health Research, Turku University Hospital and University of Turku, Finland; 4 Administrative Center, Hospital District of Southwest Finland, Turku, Finland; 5 Department of Public Health, Faculty of Medicine, University of Helsinki, Finland; 6 Research Services and Department of Clinical Medicine, Turku University Hospital, Finland; 7 Division of Geriatric Medicine, University of Turku, Finland; 8 Department of Public Health, University of Turku, Finland; 9 Turku Clinical Research Center, Turku University Hospital, Finland

**Keywords:** Cardiovascular, Health disparities, Outcomes, Quality of care

## Abstract

**Background:**

The female sex is associated with poorer outcomes after myocardial infarction (MI), although current evidence in older patients is limited and mixed. We sought to evaluate sex-based differences in outcome after MI in older patients.

**Method:**

Consecutive older (≥70 years) all-comer patients with out-of-hospital MI admitted to 20 hospitals in Finland between 2005 and 2014 were studied using national registries (*n* = 40 654, mean age 80 years, 50% women). The outcome of interest was death within 1 year after MI. Differences between sexes (age, baseline features, medication, comorbidities, revascularization, and treating hospital) were balanced by inverse probability weighting.

**Results:**

Adjusted all-cause case fatality was lower in women than in men at 30 days (16.0% vs 19.0%, respectively) and at 1 year (27.7% vs 32.4%, respectively) after MI (hazard ratio: 0.83; confidence interval [CI]: 0.80–0.86; *p* < .0001). Excess 1-year case fatality after MI compared to the corresponding general population was 22.1% (CI: 21.4%–22.8%) in women and 24.1% (CI: 23.4%–24.9%) in men. Women had a lower adjusted hazard of death after MI in subgroups of patients aged 70–79 years and ≥80 years, patients with and without ST elevation MI, revascularized and non-revascularized patients, patients with and without atrial fibrillation, and patients with and without diabetes. The sex difference in case fatality remained similar during the study period.

**Conclusions:**

Older women were found to have a lower hazard of death after an out-of-hospital MI when compared to older men with similar features and treatments. This finding was consistent in several subgroups.

Ischemic heart disease is the leading cause of death in the older population (1), with myocardial infarction (MI) being the most common fatal disease manifestation. Older patients with MI have an especially high risk of death during the acute MI and post-MI periods (2). Identification of vulnerable patient groups allows for more efficient targeting of evidence-based therapies for MI care and secondary prevention post-MI. While many diseases and their outcomes are sex-related (3), the potential influence of sex on outcomes after MI is controversial. Previous studies have associated the female sex with poorer outcomes post-MI (4–6). Women are commonly found to receive less invasive treatment (2) and less evidence-based cardiovascular medications after MI compared to men (7), which may influence sex differences in outcomes (8). In addition, potential sex differences in outcome are likely to be age-dependent (9). In the limited studies on sex differences in outcome after MI in older patients, the results are mixed, and outcomes beyond the short term are largely uncharacterized (10–12). Therefore, we aimed to further clarify potential sex-based differences in the outcome of patients aged ≥70 years after MI in a nationwide cohort study.

## Method

### Design

**Figure 1.**
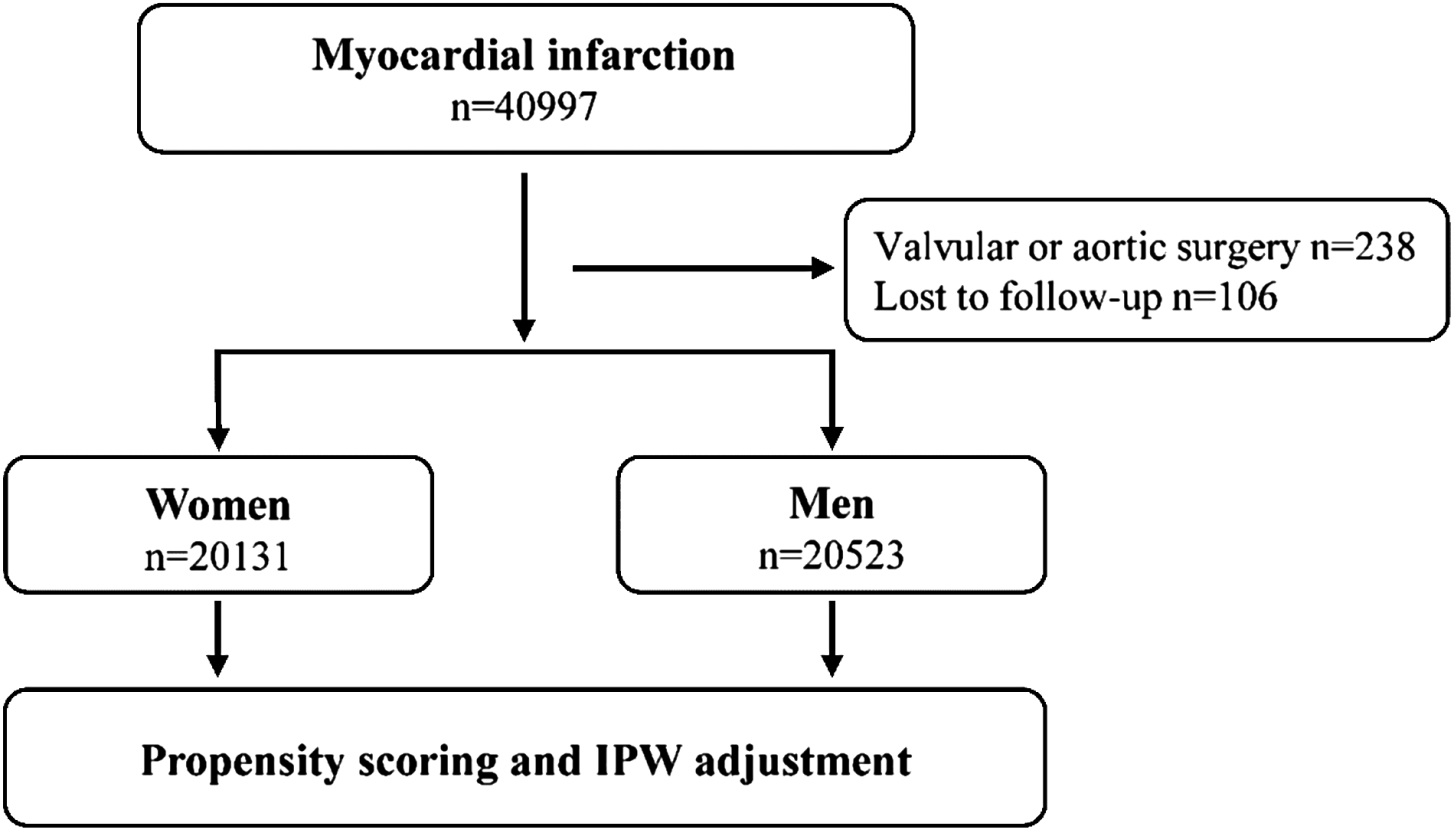
Study flowchart. IPW = inverse probability weighting.

The data of all consecutive MI patients aged ≥70 years admitted to participating hospitals in Finland between January 1, 2005 and December 31, 2014 were retrospectively collected from the Care Register for Healthcare in Finland (CRHF). All Finnish hospitals equipped with a coronary catherization laboratory (*n* = 20, including 5 university hospitals with emergency coronary surgery available) that treat MI patients were included. First-time admissions during the study period of out-of-hospital MIs (arriving through either the emergency department or paramedic services) to medical (including cardiology), surgical (including cardiac surgery), or intensive care wards were included. Patients lost to follow-up (0.3%) and those treated with non-coronary cardiac or aortic surgery were excluded (Figure 1). The index MI was identified with ICD-10 code I21 as the primary diagnosis. The outcome of interest was death within 365 days after MI. Comorbidities, procedures, and prescription medications are defined in Supplementary Tables 1 and 2. Ward and hospital transfers after MI admission were combined as a single admission.

Inverse probability weighting (IPW) was used to balance cofounding differences between the study groups. Logistic regression was used to create a propensity score based on age, alcohol abuse, anemia, atrial fibrillation, cerebrovascular disease, chronic pulmonary disease, coagulopathy, dementia, depression, heart failure, hypertension, hypothyroidism, insulin-dependent diabetes, liver disease, malignancy, non-insulin-dependent diabetes, paralysis, peripheral vascular disease, prior coronary artery bypass grafting surgery (CABG), psychotic disorder, rheumatic disease, renal failure, valvular disease, revascularization by percutaneous coronary intervention or CABG, ST elevation MI (STEMI), use of cardiovascular medications prior to MI (adenosine diphosphate inhibitors, anticoagulant, angiotensin-converting enzyme inhibitor/angiotensin II receptor blocker, aldosterone antagonists, antiarrhythmics, β-blockers, and statins), treating hospital, and study year. Stabilized IPW was then calculated using the propensity score (13). Inverse probability weighting resulted in balanced sex groups (Table 1), with a 0.005 standardized mean difference (SMD) of the propensity score. The mean of stabilized IPWs was 1.00 (*SD* 0.49). Separate IPW balancing was performed on the cohort without revascularization as a propensity variable. Subgroup analyses, with separate propensity scoring and IPW adjusting, were performed for patients aged 70–79 years and ≥80 years, patients with and without STEMI, revascularized and non-revascularized patients, patients with and without atrial fibrillation, and patients with and without diabetes. Baseline variables were balanced between the sexes in all subgroups (*p* > .419 and SMD < 0.016 for all). In addition, multivariable regression models adjusted with the same variables used for propensity scoring were studied.

**Table 1.** Baseline Features of Older Women and Men With Out-of-Hospital MI

	Nonadjusted Patients				Weighted Patients			
	Women	Men			Women	Men		
Variable	*n* = 20 131	*n* = 20 523	*p* Value	SMD	*n* = 20 239	*n* = 20 451	*p* Value	SMD
Age, y (*SD*)	81.6 (6.3)	78.7 (5.9)	<.0001	0.484	80.1 (6.4)	80.1 (6.3)	.638	0.005
Comorbidities								
Alcohol abuse	0.5%	1.9%	<.0001	0.127	1.2%	1.2%	.951	0.001
Anemia	5.9%	4.7%	<.0001	0.053	5.3%	5.4%	.653	0.004
Atrial fibrillation	25.3%	23.2%	<.0001	0.049	24.1%	24.0%	.810	0.002
Cerebrovascular disease	15.6%	16.5%	.019	0.023	16.0%	16.1%	.859	0.002
Chronic pulmonary disease	14.1%	16.9%	<.0001	0.079	15.9%	15.7%	.567	0.006
Coagulopathy	0.4%	0.4%	.411	0.008	0.4%	0.4%	.960	0.0005
Dementia	12.2%	9.0%	<.0001	0.104	10.6%	10.7%	.609	0.005
Depression	16.0%	9.1%	<.0001	0.192	10.3%	10.5%	.385	0.009
Diabetes	28.3%	27.3%	.015	0.024	27.7%	27.7%	.979	0.0003
Insulin dependent	8.7%	8.0%	.012	0.025	8.3%	8.3%	.941	0.001
Non-insulin dependent	19.6%	19.2%	.321	0.001	19.4%	19.4%	.983	0.0002
Heart failure	40.5%	31.5%	<.0001	0.187	35.7%	35.8%	.924	0.001
Hypertension	64.5%	52.5%	<.0001	0.246	58.2%	58.1%	.821	0.002
Hypothyroidism	9.5%	2.5%	<.0001	0.300	6.0%	5.9%	.907	0.001
Liver disease	0.7%	0.7%	.761	0.003	0.8%	0.8%	.631	0.005
Malignancy	12.7%	20.3%	<.0001	0.206	17.1%	16.8%	.419	0.008
Metastatic tumor	0.3%	0.4%	.451	0.007	0.3%	0.3%	.942	0.001
Paralysis	0.4%	0.4%	.797	0.003	0.4%	0.4%	.697	0.004
Peripheral vascular disease	8.9%	11.8%	<.0001	0.094	10.3%	10.3%	.822	0.002
Prior CABG	2.5%	5.5%	<.0001	0.152	4.0%	4.0%	.962	0.0004
Prior MI	20.9%	24.6%	<.0001	0.086	22.9%	22.8%	.878	0.002
Psychotic disorder	3.9%	2.4%	<.0001	0.090	3.1%	3.2%	.539	0.006
Rheumatic disease	9.6%	5.8%	<.0001	0.144	7.6%	7.6%	.943	0.001
Renal failure	4.9%	6.5%	<.0001	0.069	5.7%	5.7%	.999	<0.0001
Valvular disease	9.4%	7.5%	<.0001	0.068	8.4%	8.5%	.881	0.001
Revascularization	29.2%	42.2%	<.0001	0.274	36.0%	36.1%	.686	0.004
PCI	25.8%	35.0%	<.0001	0.202	30.8%	30.8%	.941	0.001
CABG	3.7%	7.6%	<.0001	0.173	5.8%	5.7%	.826	0.002
ST elevation MI	28.9%	31.2%	<.0001	0.048	30.0%	29.9%	.853	0.002
Anterior^a^	55.6%	53.3%	.013	0.045	54.0%	54.4%	.709	0.007
Medication								
ADP inhibitor	3.5%	4.1%	.004	0.029	3.8%	3.8%	.974	0.0003
Antiarrhythmic	0.4%	0.6%	.003	0.030	0.5%	0.5%	.740	0.003
Anticoagulant	9.1%	10.7%	<.0001	0.055	9.8%	9.9%	.811	0.002
ACEi or ARB	40.2%	34.5%	<.0001	0.118	37.2%	37.1%	.852	0.002
Aldosterone antagonist	2.9%	1.6%	<.0001	0.075	2.1%	2.2%	.740	0.003
β-Blocker	49.4%	44.9%	<.0001	0.091	46.9%	50.0%	.918	0.001
Statin	28.5%	30.3%	<.0001	0.040	29.3%	29.3%	.906	0.001
Treating hospital (*n* = 20)			<.0001	0.059			.195	0.002

*Notes*: ACEi = angiotensin-converting-enzyme inhibitor; ADP = adenosine diphosphate; ARB = angiotensin receptor blocker; CABG = coronary artery bypass grafting surgery; MI = myocardial infarction; PCI = percutaneous coronary intervention; SMD = standardized mean difference.

^a^Of ST elevation MI patients. Features of all patients and inverse probability weight balanced cohort.

### Data Sources

The CRHF registry data, including the data for all hospital admissions and major interventional procedures (14) and cancer data in the Finnish Cancer Registry, were obtained from the National Institute for Health and Welfare of Finland (permission no: THL/2245/5.05.00/2019). Fatality and population data were obtained from Statistics Finland (TK-53-484-20). Prescription medication purchase data and medication reimbursement permission data were obtained from the Social Insurance Institution of Finland (91/522/2015). The included registries are mandated by law and cover the entire Finnish population. Due to the retrospective study design, informed consent was not required, and the participants were not contacted.

### Statistical Analysis

Differences between study groups were analyzed with *t*, Jonckheere–Terpstra, and chi-squared tests. The effect sizes in the baseline characteristics between groups were evaluated by SMD. Case fatality was studied using the Kaplan–Meier method and Cox regression. The follow-up time was 1 year and included a complete follow-up of all included studied patients. Schoenfeld residuals were used for confirmation of proportional hazard assumptions. The use of revascularization was studied using logistic regression. Regression models were weighted with stabilized IPW. The *E*-value for estimating unmeasured confounding was calculated as previously described (15). Excess fatality after MI was calculated by subtracting the baseline fatality in the corresponding age-, sex-, and calendar year-specific group in the total Finnish population from case fatality after MI. The results are given as the mean, median, percentage, hazard ratio (HR), or odds ratio (OR) with a 95% confidence interval (CI), interquartile range (IQR), or ±*SD*. Statistical significance was defined as a *p* value <.05. Analyses were performed with SAS version 9.4 (SAS Institute, Inc., Cary, NC).

## Results

In this study on case fatality after MI, women were older and had a higher frequency of atrial fibrillation, diabetes, dementia, depression, heart failure, psychoses, and rheumatic diseases, while men had a higher frequency of peripheral and cerebral vascular diseases and malignancies in the nonadjusted cohort (Table 1). STEMI was more frequent in men. The differences between men and women were equalized with IPW adjustment, resulting in a balanced study population of 20 239 women and 20 451 men (Table 1; Supplementary Figure). Women were less frequently revascularized during MI admission in both absolute terms (Table 1) and when adjusting for baseline differences and medications than men (33.3% vs 38.4%, respectively; OR: 0.80; CI: 0.77–0.83; *p* < .0001). The duration of MI admission for hospital survivors was longer in women (median: 9; IQR: 6–16 days) than in men (median: 8; IQR: 5–14 days; *p* < .0001).

**Figure 2.**
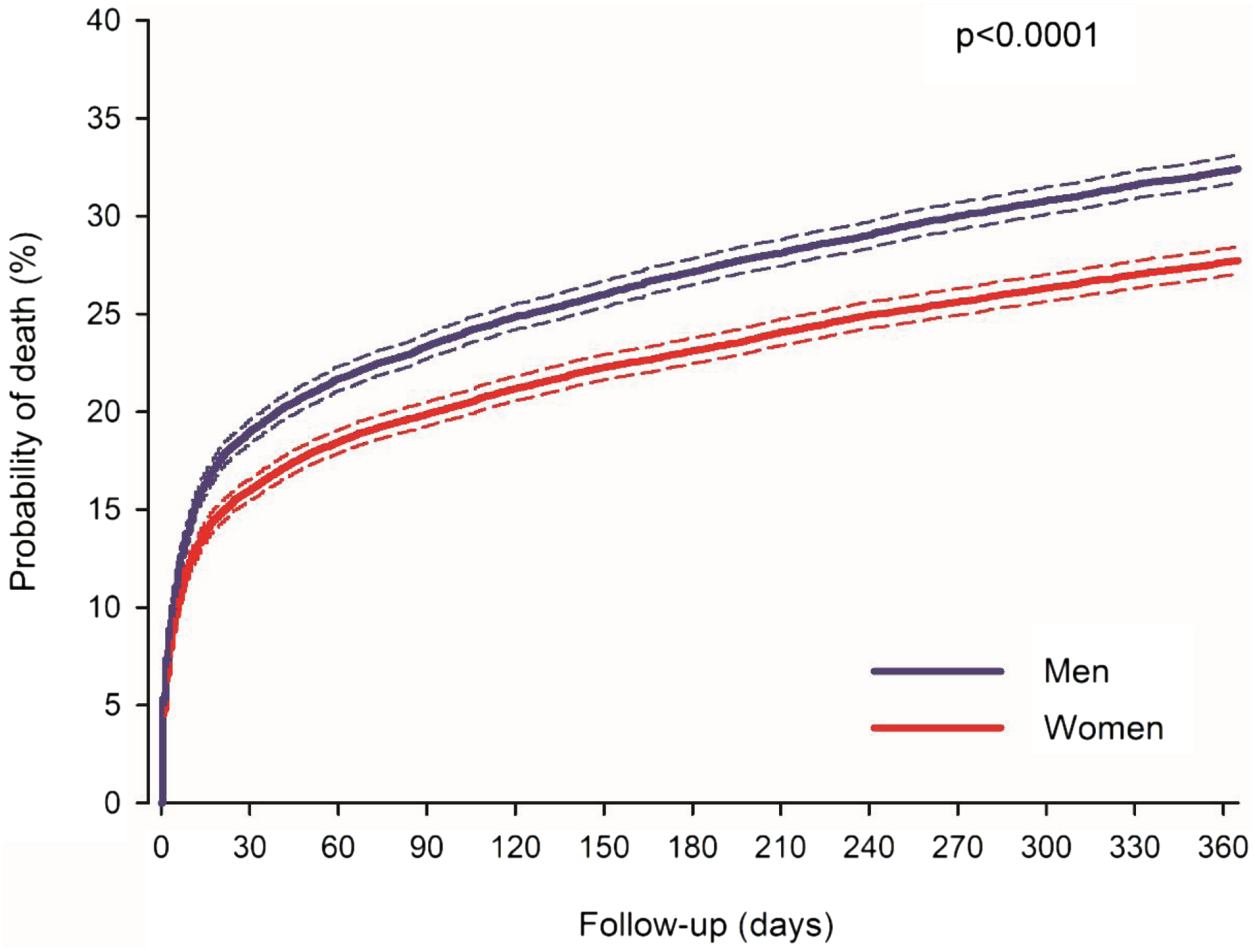
Inverse probability weight-adjusted case fatality for older women and men after myocardial infarction. Dashed lines represent 95% confidence intervals.

During the 1-year follow-up, 12 241 weighted patients died (5 612 women). Nonadjusted case fatality was higher in women at 30 days (18.2% in women vs 16.9% in men; *p* = .0003) and 1 year after MI (31.4% vs 29.1%, respectively; nonadjusted HR: 1.10; CI: 1.06–1.14; *p* < .0001) (Supplementary Table 3). However, when adjusted for baseline features, cardiovascular medication, and revascularization, case fatality was lower in women after MI (Figure 2). The IPW-adjusted 30-day case fatality was 16.0% in women versus 19.0% in men (*p* < .0001). The 1-year IPW-adjusted case fatality after MI was 27.7% in women and 32.4% in men (HR: 0.83; CI: 0.80–0.86; *p* < .0001). The *E*-value for IPW-adjusted analysis was 1.74 (CI: 1.63–1.86). Multivariable adjusted HR was 0.82 (CI: 0.79–0.85; *p* < .0001). Excess fatality was 15.5% (CI: 14.9–16.1%) in women and 18.3% (CI: 17.7–18.9%) in men at 30 days and 22.1% (CI: 21.4–22.8%) in women and 24.1% (CI: 23.4–24.9%) in men at 1 year after MI. The proportion of cardiovascular deaths was similar between the sexes in the adjusted cohort (87.0% in women and 86.5% in men; *p* = .379). Autopsies were performed on 17.9% of the deceased patients, with no difference between the sexes (*p* = .488). Regarding the deceased patients in the adjusted cohort, death occurred in either the hospital ward or another health care unit for 91.3%, at home for 5.8%, and elsewhere for 2.9%. Women were less likely to die at home than men (5.0% vs 6.5%, respectively; *p* = .0006).

**Figure 3.**
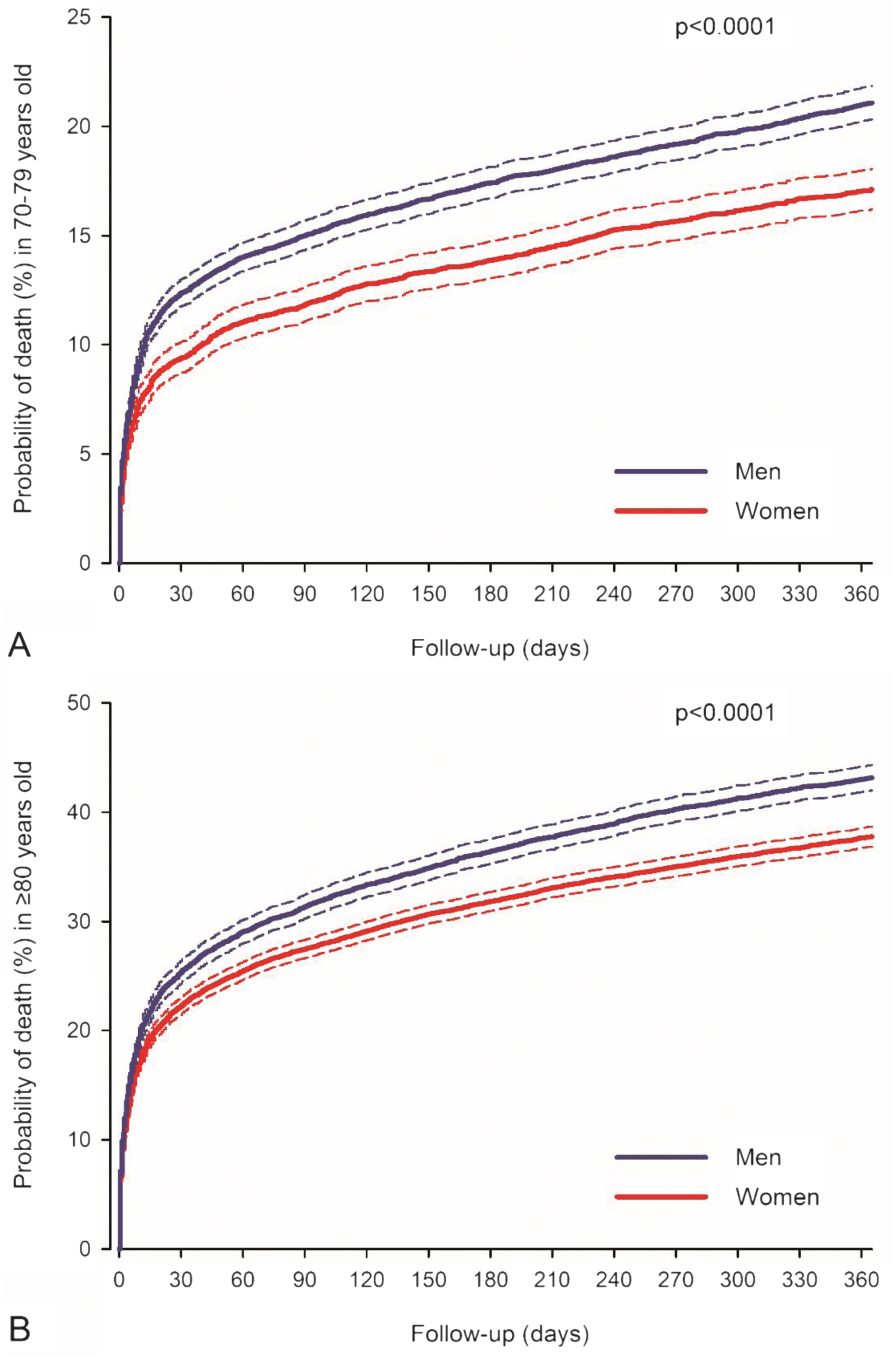
Inverse probability weight-adjusted case fatality for women and men aged 70–79 years (**A**) and ≥80 years (**B**) after myocardial infarction. Please note the scale differences in the y-axis. Dashed lines represent 95% confidence intervals.

Women had a better adjusted prognosis in all subgroups (Table 2). The sex difference in the IPW-adjusted hazard of death after MI was similar between patients aged 70–79 years and ≥80 years (Figure 3) and in subgroups of revascularized and non-revascularized patients, patients with and without atrial fibrillation, and between study eras (Table 2). Results of multivariable adjusted analyses were comparable with IPW-adjusted analyses in the subgroups (Supplementary Table 3). Although women had a better adjusted prognosis, regardless of MI presentation or diabetes status, the sex-based difference in hazard of death was higher in patients without diabetes or STEMI (Table 2).

**Table 2.** Sex-Based Inverse Probability Weight-Adjusted 30-Day and 1-Year Case Fatality in the Total Study Population and in Subgroups

	Adjusted Case Fatality			
	Women	Men	HR (95% CI)	*p* Value
All				
30 d	16.0%	19.0%		<.0001
1 y	27.7%	32.4%	0.83 (0.80–0.86)	<.0001
Year 2005–2009				
30 d	17.3%	20.4%		<.0001
1 y	30.2%	34.6%	0.85 (0.81–0.89)	<.0001
Year 2010–2014				
30 d	14.6%	17.3%		<.0001
1 y	25.0%	29.9%	0.81 (0.77–0.86)	<.0001
Age 70–79 y				
30 d	9.4%	12.4%		<.0001
1 y	17.1%	21.1%	0.79 (0.74–0.85)	<.0001
Age ≥ 80 y				
30 d	22.2%	25.3%		<.0001
1 y	37.8%	43.2%	0.84 (0.81–0.88)	<.0001
ST elevation MI				
30 d	19.6%	21.7%		.018
1 y	28.2%	31.0%	0.90 (0.84–0.96)	.001
Non-ST elevation MI				
30 d	14.3%	17.8%		<.0001
1 y	27.5%	33.0%	0.80 (0.77–0.83)	<.0001
Revascularized				
30 d	6.1%	7.0%		.072
1 y	10.6%	12.5%	0.84 (0.76–0.93)	.0005
Non-revascularized				
30 d	21.4%	25.8%		<.0001
1 y	37.3%	43.7%	0.82 (0.79–0.85)	<.0001
Atrial fibrillation				
30 d	19.2%	22.4%		.001
1 y	36.6%	41.1%	0.86 (0.81–0.92)	<.0001
No atrial fibrillation				
30 d	14.9%	17.9%		<.0001
1 y	25.0%	29.7%	0.82 (0.78–0.86)	<.0001
Diabetes				
30 d	18.9%	20.7%		.043
1 y	33.5%	36.3%	0.91 (0.85–0.96)	.002
No diabetes				
30 d	14.9%	18.3%		<.0001
1 y	25.6%	30.8%	0.80 (0.77–0.84)	<.0001

*Note*: HR = hazard ratio within 1-y follow-up; MI = myocardial infarction.

## Discussion

This population-based study investigated sex-based differences in case fatality after out-of-hospital MI in an older all-comer population of Finland. Women were found to have a significantly lower hazard of 30-day and 1-year death than men after MI admission when equalized for differences in baseline features and treatments, comorbidities, and revascularization. Sex differences in post-MI fatality were present in subgroups of patients aged 70–79 years and ≥80 years, patients with and without STEMI, revascularized and non-revascularized patients, diabetic and nondiabetic patients, and in patients with and without atrial fibrillation.

The female sex has been associated with poorer MI outcome in several studies (4–6,9), but the difference appears to be especially present in younger MI patients (9,11), while the results for older patients are more limited and mixed. In a U.S. study, Cenko et al. found no sex difference in 30-day case fatality after adjustments in STEMI patients aged >60 years (16). Another study based on U.S. SILVER-AMI data found sex not to be an independent predictor of 6-month post-MI death in patients aged ≥75 years (12). A recent study of STEMI patients aged >65 years in Arabian Gulf region registries found a higher 1-year-adjusted case fatality rate in women (17). A German registry study found that women ≥80 years of age have higher in-hospital fatality after STEMI, but no sex difference was present in younger patients, and women had a better prognosis after a non-STEMI had occurred (18). A Swedish study by Berg et al. using data registry data similar to ours found women aged 75–84 years have a lower 28-day case fatality after MI admission, while no sex difference was observed in women aged 65–74 years (11). However, another Swedish study with SWEDEHEART registry data found women to have excess 1-year fatality compared to men after MI, which increased with aging (19). Despite these seemingly contradictory results, nonadjusted fatality after MI was found to be higher in women in almost all relevant studies, including the current one. Older women with MI are less likely to receive evidence-based revascularization and medications than men, as shown by our data and by previous investigations (8,20). Our results, when combined with those of previous studies, show a pattern of improving prognosis in women with increasing adjustments for sex differences.

Our IPW-adjusted results strongly suggest that being male is an independent predictor of death after MI in the older population, although regional and health care system-related differences are naturally possible (21).

MI patients are a heterogeneous population with many factors as outcomes. Adequate revascularization improves MI outcomes, regardless of the patient’s age, but it is less commonly used in older patients, which is partly due to comorbidities and complication risks (22). Atrial fibrillation patients (24% of our study population) are at a higher bleeding risk due to anticoagulation, and they have type II MIs more frequently (23). Older diabetic patients generally have more severe coronary disease, higher comorbidity burden, and poorer prognosis after MI (24). Patients that suffer a STEMI require immediate revascularization due to coronary artery occlusion, while those without ST elevation are a more heterogeneous group and may have a poorer prognosis (22). Older age increases complications after MI, with patients ≥80 years of age having significantly poorer outcomes than those aged 70–79 years (2). Notably, we found the baseline feature-adjusted sex difference in case fatality after MI to be present in all these patient subgroups. However, the sex difference was larger in nondiabetics and in non-STEMI patients. This may relate to a poorer baseline prognosis in diabetics and possibly to sex differences regarding the extent of coronary artery disease in non-STEMI patients (25). Older men have a higher baseline fatality than women, influencing true MI-related sex differences at follow-up (18). In our data, the death rate adjusted for excess case fatality was also higher in men, underlying a poorer prognosis for men. Sex disparities in outcome after MI are reported to be attenuated over time (9), but in agreement with a previous Swedish study (11), we found no significant change in sex difference of adjusted fatality over time. Sex-related outcome trends may be associated with temporal changes in treatment and risk factor management (26).

The current study provides evidence for sex differences in post-MI outcomes after balancing for multiple sex differences in baseline features and treatment. However, uncovering effectors of this sex discrepancy is beyond the current study and must be further clarified in a future study. Potential contributors may include biological as well as behavioral and psychosocial factors, such as more frequent high-risk lifestyles and less frequent help-seeking behaviors for health problems in men (27). While older women are more likely to feel lonely, older men experiencing loneliness are known to be at greater risk of death (28). It has been proposed that men are more prone to acquiring life-threatening conditions compared with women (27). Even in our nonadjusted cohort, many of the chronic conditions were more frequent in women, but serious conditions, such as malignancies and cerebrovascular diseases, were more frequent in men.

Frailty and functional impairment are associated with poorer outcomes after MI (29). Although we did not directly measure frailty, the studied comorbidities include major contributors to the previously validated frailty risk score (30). Given that older women have a higher prevalence of frailty (31) and functional impairment (20), it seems unlikely that the observed sex difference would be explainable by nondetected frailty or functional impairment. A possible underlying explanation for the sex difference observed in the present study could be related to a phenomenon known as the “male–female health-survival paradox” (32): while with advancing age, more women than men are living with frailty, the risk of dying is higher in men. Recently, the phenomenon has been extended to include older acutely hospitalized populations (33). The potential role of frailty on the sex difference observed here in the case fatality after MI in older patients warrants to be addressed in further studies.

Our findings have important implications for treatment and secondary prevention after MI in older patients. Widespread, intensive, non-sex-dependent usage of evidence-based MI treatments and post-MI secondary prevention are necessary for equal, ethically sound, optimal care of the increasing older population with coronary artery disease. In addition, our results imply that special attention should be paid to older men after MI. However, we are not in any way suggesting poorer or less invasive MI treatments for women.

There are strengths and limitations to the current study. We used a combination of nationwide, previously validated, mandatory-by-law registries (34) and conducted a complete follow-up review. Analyses were adjusted with broader coverage of confounders than previous registry studies on the subject. Furthermore, the main result of the study was consistent in sensitivity analyses of various subgroups. Propensity scoring and IPW, which we used, are among the strongest methods for controlling confounding factors in comparative registry studies. This methodology allows straightforward presentation of results similar to randomized trials while providing noninferior or superior bias reduction compared to multivariable regression (35). Of note, IPW-adjusted results were virtually equal to those of conventional multivariable covariate adjustment in our study. The advantage of IPW compared to propensity matching is the usage of all available data for analysis (13,35).

Nonrecognized residual confounders are nevertheless possible. Retrospective use of registry data without access to more detailed laboratory, imaging, socioeconomical, or lifestyle information is a major inherent limitation of registry studies, including the current one. Based on the *E*-value, the observed higher adjusted case fatality in men could be explained away by an unmeasured confounder that was associated with both sex and fatality by a risk ratio of ≥1.7 each, above and beyond the measured confounders (15). Considering the extent of studied confounders in an all-comer population-based setting this seems however unlikely. Diagnoses in registries were made by treating physicians, coding errors could have occurred, and underreporting of medical history data is possible. However, it is unlikely that these limitations would influence women and men differently and thus significantly affect the main finding of the study.

## Conclusion

We found that older women have a significantly lower hazard of death after admission for out-of-hospital MI than older men with similar features and treatments. The results were consistent in several subgroups, including patients aged ≥80 years, STEMI patients, revascularized patients, diabetic patients, and patients with atrial fibrillation. Evidence-based application and equality in the implementation of treatments for MI and secondary prevention afterward are prerequisites for improving the outcome and well-being of the growing older population with coronary artery disease. Our results also imply that special attention should be paid to older men after MI.

## Supplementary Material

glab152_suppl_Supplementary_material
