# Supplementary material for: Sex Difference in the Case Fatality of Older Myocardial Infarction Patients
Source: J Gerontol A Biol Sci Med Sci. 2021 May 28;77(3):614–20. doi: 10.1093/gerona/glab152 (PMC8893190; doi:10.1093/gerona/glab152)
Supplement: glab152_suppl_Supplementary_material [file glab152_suppl_supplementary_material.docx]

**Supplement**

**Sex Difference in the Case Fatality of Older Myocardial Infarction Patients**

Ville Kytö, M.D. Ph.D.^1,2,3,4,5^, Maria Nuotio, M.D. Ph.D.^6,7^, Päivi Rautava, M.D. Ph.D.^8,9^

^1^Heart Center, Turku University Hospital and University of Turku, Turku, Finland

^2^Research Center of Applied and Preventive Cardiovascular Medicine, University of Turku, Turku, Finland

^3^Center for Population Health Research, Turku University Hospital and University of Turku, Turku, Finland

^4^Administrative Center, Hospital District of Southwest Finland, Turku, Finland

^5^Department of Public Health, Faculty of Medicine, University of Helsinki, Helsinki, Finland

^6^Research Services and Department of Clinical Medicine, Turku University Hospital, Turku, Finland

^7^Division of Geriatric Medicine, University of Turku, Turku, Finland

^8^Department of Public Health, University of Turku, Turku, Finland

^9^Turku Clinical Research Center, Turku University Hospital, Turku, Finland

Correspondence:

Ville Kytö, M.D. Ph.D.

Heart Center, Turku University Hospital

P.O. Box 52, 20521 Turku, Finland

Tel.: +358 2 3130000

email: [ville.kyto@utu.fi](mailto:ville.kyto@utu.fi)

|  | | **ICD-10 codes** | **Prescription drug reimbursement codes** | **Operational codes** |
| --- | --- | --- | --- | --- |
| **Alcohol abuse** | | F10, E52, G62.1, I42.6, K29.2, K70.0, K70.3, K70.9, T51, Z50.2, Z71.4, Z72.1 | 309 |  |
| **Anemia** | | D50.0, D50.8, D50.9, D51-D53 | 107, 122,175, 376 |  |
| **Atrial fibrillation** | | I48 |  |  |
| **Cerebrovascular disease** | | G45, G46, H34.0, I60–I69 |  |  |
| **Chronic pulmonary disease** | | I27.8, I27.9, J40–J47, J60–J67, J68.4, J70.1, J70.3 | 203, 210, 284, 304, 348, 356 |  |
| **Coagulopathy** | | D65–D68, D69.1, D69.3– D69.6 | 126, 161, 172, 194, 332, 357, 375 |  |
| **Dementia** | | A81.0, F00–F03, F05.1, G30, G31 | 307 |  |
| **Depression*** | | F20.4, F31.3–F31.5, F32, F33, F34.1, F41.2, F43.2 |  |  |
| **Diabetes**** | | E10-E14 | 103, 215, 160, 162, 358, 171, 177, 285, 346, 371, 382 |  |
| **Heart failure** | | I09.9, I11.0, I13.0, I13.2, I25.5, I42.0, I42.5–I42.9, I43, I50, P29.0 | 201, 283, 354, 381 |  |
| **Hypertension** | | I10-I13, I15 | 205 |  |
| **Hypothyroidism** | | E00-E03, E89.0 | 104 |  |
| **Liver disease** | | B18, I85, I86.4, I98.2, K70, K71.1, K71.3– K71.5, K71.7, K72– K74, K76.0, K76.2– K76.9, Z94.4 |  |  |
| **Malignancy** | | C | 115, 116, 128, 130, 155, 156, 158, 159, 163, 167, 168, 169, 173, 176, 180, 184, 185, 189, 191, 192, 197, 198, 302, 311, 312, 322, 323, 324, 325, 328, 329, 337, 340, 341, 343, 352, 360, 361, 362, 363, 369, 372, 373, 378 |  |
|  | **Metastatic tumor** | C77-C80 |  |  |
| **Paralysis** | | G04.1, G11.4, G80.1, G80.2, G81, G82, G83.0–G83.4, G83.9 |  |  |
| **Peripheral vascular disease** | | I70, I71, I73.1, I73.8, I73.9, I77.1, I79.0, I79.2, K55.1, K55.8, K55.9, Z95.8, Z95.9 |  |  |
| **Prior CABG** | | Z951 |  | FNA, FNB, FNC, FND, FNE*** |
| **Prior myocardial infarction** | | I21***, I22, I25.2 |  |  |
| **Psychotic disorder** | | F20, F21–F25, F28, F29, F30.2, F31.2, F31.5 | 112, 188 |  |
| **Rheumatic disease** | | L94.0, L94.1, L94.3, M05, M06, M08, M12.0, M12.3, M30, M31.0– M31.3, M32–M35, M45, M46.0, M46.1, M46.4, M46.8, M46.9 | 202, 281 |  |
| **Renal failure** | | I12.0, I13.0, N18, N19, N25.0, Z49.0– Z49.2, Z94.0, Z99.2 | 137, 138, 320 |  |
| **Valvular disease** | | A52.0, I05–I08, I09.1, I09.8, I34–I39, Q23.0–Q23.3, Z95.2– Z95.4 |  |  |
| **Revascularization** | |  |  |  |
|  | **PCI** |  |  | FN2AA, FN2AB, FN2BA, FN2CA, FN2CB, FN2CC, FN2CD, FN2CE, FN2CT, FN2DA, FN2DB, FN2EA, FN1AT, FN1BT, FN1YT, FNG00, FNG10, FN2DA, TFN40, TFN50, TFN10, FN2EF, FN2EB, FN2EC, FN1ST |
|  | **CABG** |  |  | FNA, FNB, FNC, FND, FNE |
| **ST-elevation MI** | | I21.0-I21.3 |  |  |
|  | **Anterior** | I21.0 |  |  |

* Or purchase of antidepressant medication within 90 days preceding index admission. ** Or purchase of antidiabetic medication within 90 days preceding index admission. *** Only preceding index admission.

**Supplement Table 1.** Definitions for co-morbidities and patient baseline features. International Classification of Diseases (ICD) coding adopted with minor modifications from Quan H, Sundararajan V, Halfon P, Fong A, Burnand B, Luthi JC, Saunders LD, Beck CA, Feasby TE, Ghali WA. Coding Algorithms for Defining Comorbidities in ICD-9-CM and ICD-10 Administrative Data. Med Care 2005;43: 1130–1139.

|  | | **ATC-codes** |
| --- | --- | --- |
| **ADP-inhibitor** | | B01AC04, B01AC05, B01AC22, B01AC24 |
| **Anticoagulant** | | B01AA, B01AE, B01AF, B01AX06 |
| **Antidiabetic** | | A10 |
|  | **Insulin** | A10A |
|  | **Non-insulin** | A10B |
| **ACEi or ARB** | | C09, C10BX04, C10BX06, C10BX07, C10BX10, C10BX11, C10BX12, C10BX13, C10BX14, C10BX15 |
| **Aldosterone antagonist** | | C03DA01, C03DA04 |
| **Antiarrhythmic** | | C01B |
| **Beta-blocker** | | C07, C09BB, C09DA, C09BX02, C09DX05 |
| **Statin** | | C10AA, C10BA, C10BX |

**Supplement Table 2.** Anatomical Therapeutic Chemical Classification (ATC) codes for prescription medications. ACEi = angiotensin-converting-enzyme inhibitor, ADP = adenosine diphosphate, ARB = angiotensin receptor blocker. Usage of medication was defined as purchase within 90 days preceding index admission.

|  | | **Non-adjusted case-fatality** | | **Univariable** | | **Multivariable** | |
| --- | --- | --- | --- | --- | --- | --- | --- |
|  | | **Women** | **Men** | **HR (95%CI)** | ***P*-value** | **HR (95%CI)** | ***P*-value** |
| **All** | |  |  |  |  |  |  |
|  | 30-day | 18.2% | 16.9% |  | 0.0003 |  | <0.0001 |
|  | 1-year | 31.4% | 29.1% | 1.10 (1.06-1.14) | <0.0001 | 0.82 (0.79-0.85) | <0.0001 |
| **Year 2005-2009** | |  |  |  |  |  |  |
|  | 30-day | 19.2% | 18.6% |  | 0.250 |  | <0.0001 |
|  | 1-year | 33.5% | 31.4% | 1.08 (0.13-1.13) | 0.002 | 0.83 (0.79-0.87) | <0.0001 |
| **Year 2010-2014** | |  |  |  |  |  |  |
|  | 30-day | 17.0% | 15.0% |  | 0.0002 |  | <0.0001 |
|  | 1-year | 28.8% | 26.6% | 1.11 (1.05-1.17) | 0.0002 | 0.81 (0.76-0.86) | <0.0001 |
| **Age 70-79 years** | |  |  |  |  |  |  |
|  | 30-day | 9.9% | 11.9% |  | <0.0001 |  | <0.0001 |
|  | 1-year | 18.0% | 20.4% | 0.87 (0.82-0.93) | <0.0001 | 0.77 (0.72-0.83) | <0.0001 |
| **Age ≥80 years** | |  |  |  |  |  |  |
|  | 30-day | 23.2% | 23.7% |  | 0.444 |  | <0.0001 |
|  | 1-year | 39.5% | 41.1% | 0.96 (0.92-1.00) | 0.039 | 0.84 (0.80-0.88) | <0.0001 |
| **ST-elevation MI** | |  |  |  |  |  |  |
|  | 30-day | 22.8% | 18.8% |  | <0.0001 |  | 0.006 |
|  | 1-year | 32.4% | 27.4% | 1.23 (1.15-1.31) | <0.0001 | 0.87 (0.81-0.94) | 0.0002 |
| **Non-ST-elevation MI** | |  |  |  |  |  |  |
|  | 30-day | 16.3% | 16.0% |  | 0.501 |  | <0.0001 |
|  | 1-year | 31.0% | 29.8% | 1.05 (1.00-1.09) | 0.040 | 0.79 (0.76-0.83) | <0.0001 |
| **Revascularized** | |  |  |  |  |  |  |
|  | 30-day | 6.9% | 6.4% |  | 0.161 |  | 0.030 |
|  | 1-year | 11.9% | 11.7% | 1.02 (0.93-1.12) | 0.707 | 0.84 (0.76-0.93) | 0.001 |
| **Non-revascularized** | |  |  |  |  |  |  |
|  | 30-day | 22.8% | 24.5% |  | 0.002 |  | <0.0001 |
|  | 1-year | 39.4% | 41.7% | 0.93 (0.90-0.97) | 0.0002 | 0.81 (0.78-0.85) | <0.0001 |
| **Atrial fibrillation** | |  |  |  |  |  |  |
|  | 30-day | 21.3% | 20.2% |  | 0.200 |  | 0.0006 |
|  | 1-year | 39.4% | 37.7% | 1.06 (1.00-1.13) | 0.068 | 0.85 (0.79-0.91) | <0.0001 |
| **No atrial fibrillation** | |  |  |  |  |  |  |
|  | 30-day | 17.1% | 15.9% |  | 0.002 |  | <0.0001 |
|  | 1-year | 28.7% | 26.5% | 1.10 (1.06-1.15) | <0.0001 | 0.81 (0.77-0.85) | <0.0001 |
| **Diabetes** | |  |  |  |  |  |  |
|  | 30-day | 20.7% | 19.1% |  | 0.024 |  | 0.012 |
|  | 1-year | 36.7% | 33.5% | 1.12 (1.05-1.19) | 0.0004 | 0.89 (0.84-0.96) | 0.001 |
| **No diabetes** | |  |  |  |  |  |  |
|  | 30-day | 17.2% | 16.0% |  | 0.007 |  | <0.0001 |
|  | 1-year | 29.3% | 27.4% | 1.08 (1.04-1.13) | 0.0002 | 0.79 (0.75-0.83) | <0.0001 |

**Supplement Table 3.** Non-adjusted sex-based 30-day and 1-year case fatality in the total study population and in subgroups. Results of unadjusted and multivariable adjusted Cox-models (see methods for details). HR = hazard ratio within 1-year follow-up. MI = myocardial infarction.


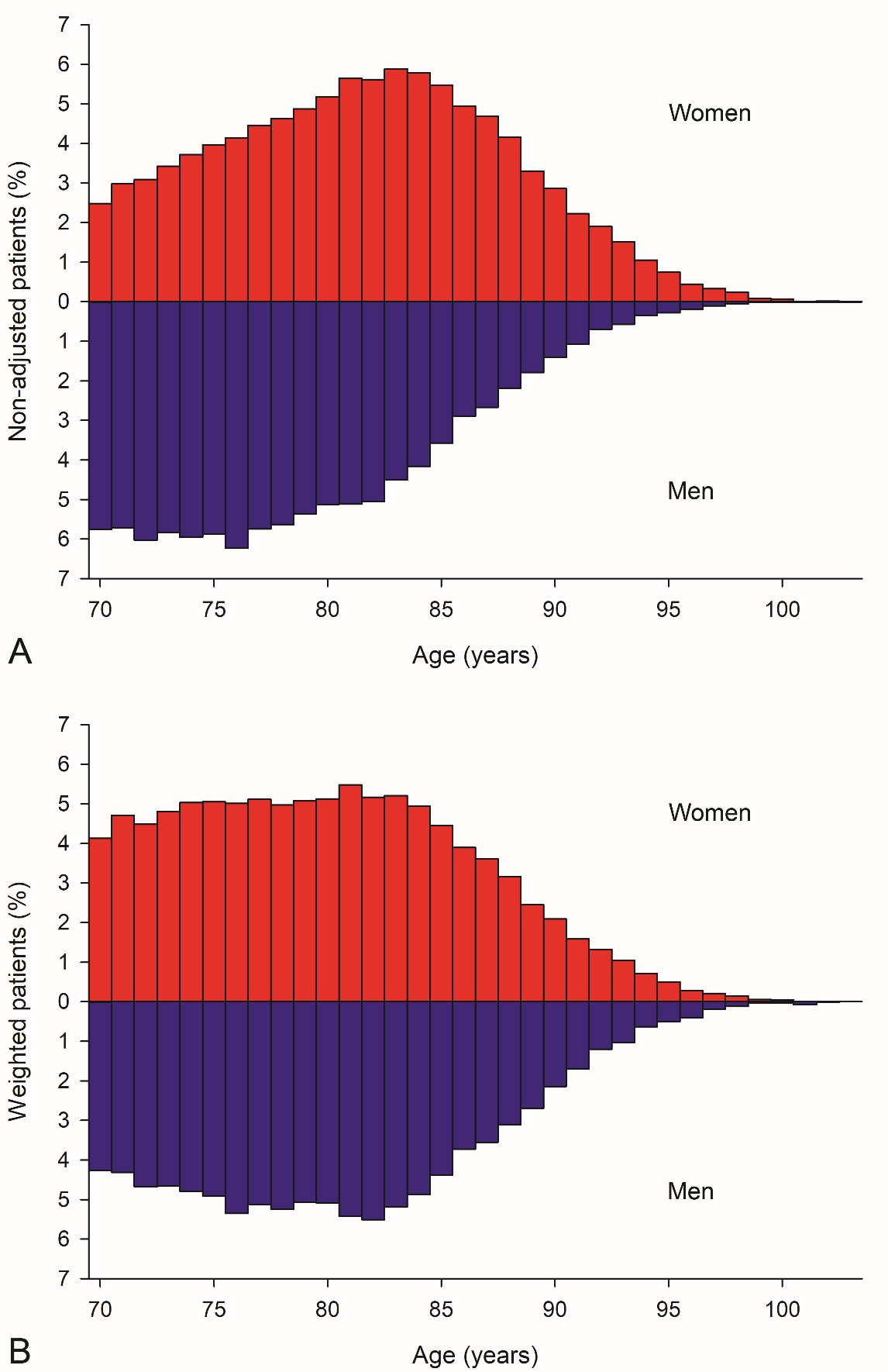


**Supplement Figure.** Age distribution of non-adjusted (A) and IPW weighted patients (B) by sex.
